# Pausing as a mechanism of nucleosome recovery

**DOI:** 10.1186/1756-8935-6-S1-P14

**Published:** 2013-03-18

**Authors:** Han-Wen Chang, Olga I Kulaeva, Alexey Shaytan, M Kibanov, K Severinov, David J Clark, Vasily M Studitsky

**Affiliations:** 1 Department of Pharmacology, UMDNJ-Robert Wood Johnson Medical School, Piscataway, NJ 08854, USA and Faculty of Biology; 2 Moscow State University; Moscow, Russia; 3 Waksman Institute of Microbiology and Department of Molecular Biology and Biochemistry, Rutgers, State University of New Jersey, Piscataway, NJ08854, USA; 4 Program in Genomics of Differentiation, Eunice Kennedy Shriver National Institute of Child Health and Human Development, National Institutes of Health, Bethesda, MD 20892, USA

## Background

Nucleosome survival during transcription is important for the maintenance of chromatin integrity, gene regulation, cell survival, and aging. In according to the studies *in vitro* and *in vivo*, Pol II pauses at positions inside of a nucleosome and nucleosome survives after Pol II transcription through chromatin [1]. A key intermediate, Ø-loop (EC+49), formed at +45 region inside of nucleosome and Pol II pausing at +45 nucleosomal region positively correlated to nucleosome survival [2,3]. A putative electrostatic interaction between Pol II and histone octamer has also been predicted to stabilize the Ø-loop intermediate by structural modeling[3]. In this study, we will provide more evidences to link those characteristics together.

## Materials and methods

Protein purification [3][4]

Computational structure modeling [3]

Transcription assay, Nucleosome fate and DNase I footprinting [3,5]

## Results

In structural modeling, three negatively charged surfaces (regions 1, 2, 3) were identified on Rpb1, a large subunit of yeast Pol II, and plausibly interacted to histone octamer, especially H2B N-tail region, by the electrostatic force. Results of transcriptions by *Thermus thermophilus* (*T.th.*) and *Thermus aquaticus* (*T.aq.*) RNAPs which contained less net negative charges at all three regions showed lower +45 pausing, no Ø-loop formation and nucleosome displacement. Finally, lower nucleosomal barrier was also shown during the transcription by mutated yeast Pol II which contains less negatively charged at region 2 of Rpb1.

## Conclusions

Collectively, we demonstrated the positive correlations of higher negative net charge of the interacting region of RNAP, the stronger +45 barrier, more efficient nucleosome survival and more efficient Ø-loop intermediate formation during transcription.
